# Quantifying functional redundancy in polysaccharide-degrading prokaryotic communities

**DOI:** 10.1186/s40168-024-01838-5

**Published:** 2024-07-02

**Authors:** Dan-dan Li, Jianing Wang, Yiru Jiang, Peng Zhang, Ya Liu, Yue-zhong Li, Zheng Zhang

**Affiliations:** 1 State Key Laboratory of Microbial Technology, Institute of Microbial Technology, Shandong University, Qingdao, 266237 China; 2 Institute of Marine Science and Technology, Shandong University, Qingdao, 266237 China

**Keywords:** Functional redundancy, Within-community, Between-community, Quantification, Prokaryotic communities, Community diversity, Glycoside hydrolase

## Abstract

**Background:**

Functional redundancy (FR) is widely present, but there is no consensus on its formation process and influencing factors. Taxonomically distinct microorganisms possessing genes for the same function in a community lead to within-community FR, and distinct assemblies of microorganisms in different communities playing the same functional roles are termed between-community FR. We proposed two formulas to respectively quantify the degree of functional redundancy within and between communities and analyzed the FR degrees of carbohydrate degradation functions in global environment samples using the genetic information of glycoside hydrolases (GHs) encoded by prokaryotes.

**Results:**

Our results revealed that GHs are each encoded by multiple taxonomically distinct prokaryotes within a community, and the enzyme-encoding prokaryotes are further distinct between almost any community pairs. The within- and between-FR degrees are primarily affected by the alpha and beta community diversities, respectively, and are also affected by environmental factors (e.g., pH, temperature, and salinity). The FR degree of the prokaryotic community is determined by deterministic factors.

**Conclusions:**

We conclude that the functional redundancy of GHs is a stabilized community characteristic. This study helps to determine the FR formation process and influencing factors and provides new insights into the relationships between prokaryotic community biodiversity and ecosystem functions.

Video Abstract

**Supplementary Information:**

The online version contains supplementary material available at 10.1186/s40168-024-01838-5.

## Background

The biogeochemical reactions are driven by a limited set of energy-converting metabolic pathways, and each of the pathways exists in various microbial clades [1, 2]. A metabolic function is usually decoupled from the assemblage of functional species, implying ‘functional redundancy’ (FR) in a microbial community [3–7]. Many coexisting but taxonomically distinct microbes encode the same energy-producing metabolic functions, and the functional redundancy within a community seems inconsistent with the expectation that species should occupy different metabolic niches. Furthermore, pathway-encoding microbes are taxonomically diverse across space or time with little effect on metabolic function, and this taxonomic variability is often thought to result from ecological drift between equivalent organisms. A central debate in microbial ecology is how and which conditions lead to the decoupling and what determines the FR degree in microbial communities [8].

Prokaryotes are the most ancient, diverse, and widespread form of life on Earth and have shaped Earth’s surface chemistry over billions of years [9]. FR widely exists in prokaryotic communities in various environments, e.g., soil, ocean, human, and plant [10–14]. The stability of microbiomes is the key to understanding climate change, including resistance, resilience, and FR [15]. Functional features may be resistant or resilient under stress even if taxonomic features change. For example, drought-wetting cycles significantly alter community composition, but functions such as carbon cycling remain resilient [16, 17]. The greater FR a soil community exhibits, the more resilient it will be after perturbations [18]. Similarly, the taxonomic composition of human microbiomes varies across individuals, but the functional capacity or gene composition of the microbiomes is highly conserved, implying significant FR [19–21]. Recent studies also showed that a high FR degree of the prokaryotic communities in the human gut increased the barrier to the engraftment of exogenous microbiota [22]. FR underlies the resilience and stability of the human microbiome in response to perturbations [23, 24]. However, the FR degree, formation process, and influencing factors in various environments are unclear.

Carbohydrates account for approximately 75% of the total biomass on Earth [25, 26]. It has been observed that the hydrolysis of complex polysaccharide cellulose in a methanogenic digester could be achieved simultaneously by dozens of different organisms [27]. Experimental evidence suggests that cellulolytic bacterial species with redundant functions play an integral role in ecosystems [28]. Carbohydrate degradation mainly relies on glycoside hydrolases (GHs), and glycan diversity promotes the evolution of GHs into many types [29–32]. Recently, we evaluated the distributions of cellulose-, xylan-, and chitin-degrading enzymes across global prokaryotic communities [33]. In this study, we developed two methods to respectively quantify the degree of functional redundancy, represented with GHs, within and between communities, and further explored the influencing factors through a large-scale analysis of prokaryotic community genetic information on a global scale.

## Materials and methods

### Acquisition of genetic information of prokaryotic communities

The EMP is a meta-analysis resource that spans a wide range of biotic and abiotic factors, physicochemical properties, and geographic locations, aiming to enhance our understanding of the organizing biogeographic principles that shape the structure of microbial communities on Earth [34]. Our analysis was performed using a subset of 10,000 samples released by the EMP, which was deemed representative across different types of environments and various studies. In addition, 5000 sequences were randomly selected from each sample. A total of 262,011 operational taxonomic units (OTUs) were acquired from the 10,000 EMP samples utilizing Deblur software [35]. Chimera filtering relied on the EMP project.

A total of 17,923 complete genomes (17,538 bacterial and 385 archaeal genomes) were collected from the RefSeq database [36]. Alignments between the EMP OTUs and prokaryotic genomes were performed using BLASTn [37, 38], and the corresponding relationship was determined with a 16S rRNA (V4 region) identity of no less than 97% as the standard. In addition, taxonomic classification information was also obtained from the NCBI Taxonomy database [39].

### Global distribution of glycoside hydrolases

GHs (EC 3.2.1.-), a broadly distributed set of enzymes, are responsible for breaking down the glycosidic linkage between multiple carbohydrates or between a carbohydrate and a non-carbohydrate component. Information pertaining to the classification of GHs was acquired from the carbohydrate-active enzyme (CAZy) database [40, 41]. Based on the classification and annotation of the CAZy database, the majority of GHs in the families GH5, GH6, GH7, GH8, GH12, GH44, GH45, and GH48 act on cellulose, while the families GH10, GH11, and GH30 are primarily associated with xylanases, and the families GH18, GH19, and GH85 are predominantly chitinases [41–43].

The number of 16S rRNA genes and the number of GH genes in the genome were counted for the OTUs corresponding to the RefSeq genomes in each EMP sample. Then, the abundance of GH genes in each sample was calculated based on the aforementioned OTUs, which are shown by the gene/cell value, that is, the average number of GH genes carried by each prokaryotic cell in the community. GH gene abundances were calculated for each family, cellulases, xylanases, and chitinases.

### Definition of functional redundancy index

To quantitatively assess the degree of FR within and between communities for each GH function, we calculated the abundance of GH genes carried by each OTU in prokaryotic communities. For a specific function, the within-community functional redundancy index FRI_a_ was calculated as

$${\text{FRI}}_{a}=-\sum {P}_{i}\text{ln}{P}_{i}$$

where ${P}_{i}$ is the relative frequency of the functional genes encoded by a specific taxon in the community (the number of genes associated with the taxon divided by the total number of genes for that function). The FRI_a_ value was used to quantify the diversity of prokaryotes encoding the same metabolic function within the community and was calculated based on the Shannon index, considering the richness and evenness of all taxa with the same metabolic function in the community.

For a specific function, the between-community functional redundancy index FRI_b_ was calculated as:

$${\text{FRI}}_{b}=1-2\frac{\sum min\left({P}_{A,i},{P}_{B,i}\right)}{\sum {P}_{A,i}+\sum {P}_{B,i}}$$

where ${P}_{A,i}$ and ${P}_{B,i}$ represent the relative frequencies of genes encoding this function in specific taxa in community A and community B, respectively. The FRI_b_ value was calculated according to the Bray–Curtis index, which is used to quantify differences in the identity of taxa encoding the same metabolic function between communities.

### Determination of environmental types, environmental factors, and community diversity

All samples were also divided into environments for comparative analysis. The EMPO classified 17 microbial environments (level 3) as free-living or host-associated (level 1) and saline or non-saline (if free-living) or animal or plant (if host-associated) (level 2) [34]. The hypersaline (saline) environment was excluded due to few samples.

The alpha diversity indices included observed OTUs, Shannon index, Chao1 index, and Faith’s PD value [44–46]. The beta diversity was represented by the Bray–Curtis index and calculated based on 16 environmental types [47]. Community latitude (deg), longitude (deg), elevation (m), temperature (deg_c), pH, and salinity (psu) values were taken from EMP-associated metadata [34].

### Functional redundancy of GHs in metagenomes

We selected 27 sediment and water microbial communities previously studied from the Yellow River Delta [48]. Using 16S rRNA amplicon sequencing data, metagenomic data, and environmental factor information, we conducted a comprehensive analysis of the FR of GHs within and between communities, along with their influencing factors. Environmental factors, alpha diversity indices, and community compositions were derived from our prior study [48]. The main workflow for metagenomic sequencing analysis involved initial quality control of sequences using Trimmomatic [49], followed by assembly of effective sequence files using Megahit [50] to obtain scaffold sequences. Subsequently, gene prediction was carried out using Prodigal [51], and non-redundant gene sets were obtained using CD-HIT [50]. Quantitative gene analysis was performed using Salmon [52] to obtain abundance information for each gene, represented as transcripts per million. Finally, gene annotation was conducted by aligning with databases using Diamond [53], yielding the composition and abundance information of GHs in each sample, which were employed to analyze the functional redundancy within and between communities using the similar methods described above for EMP data.

## Results

### Quantification of functional redundancy of prokaryotic communities

**Fig. 1:**
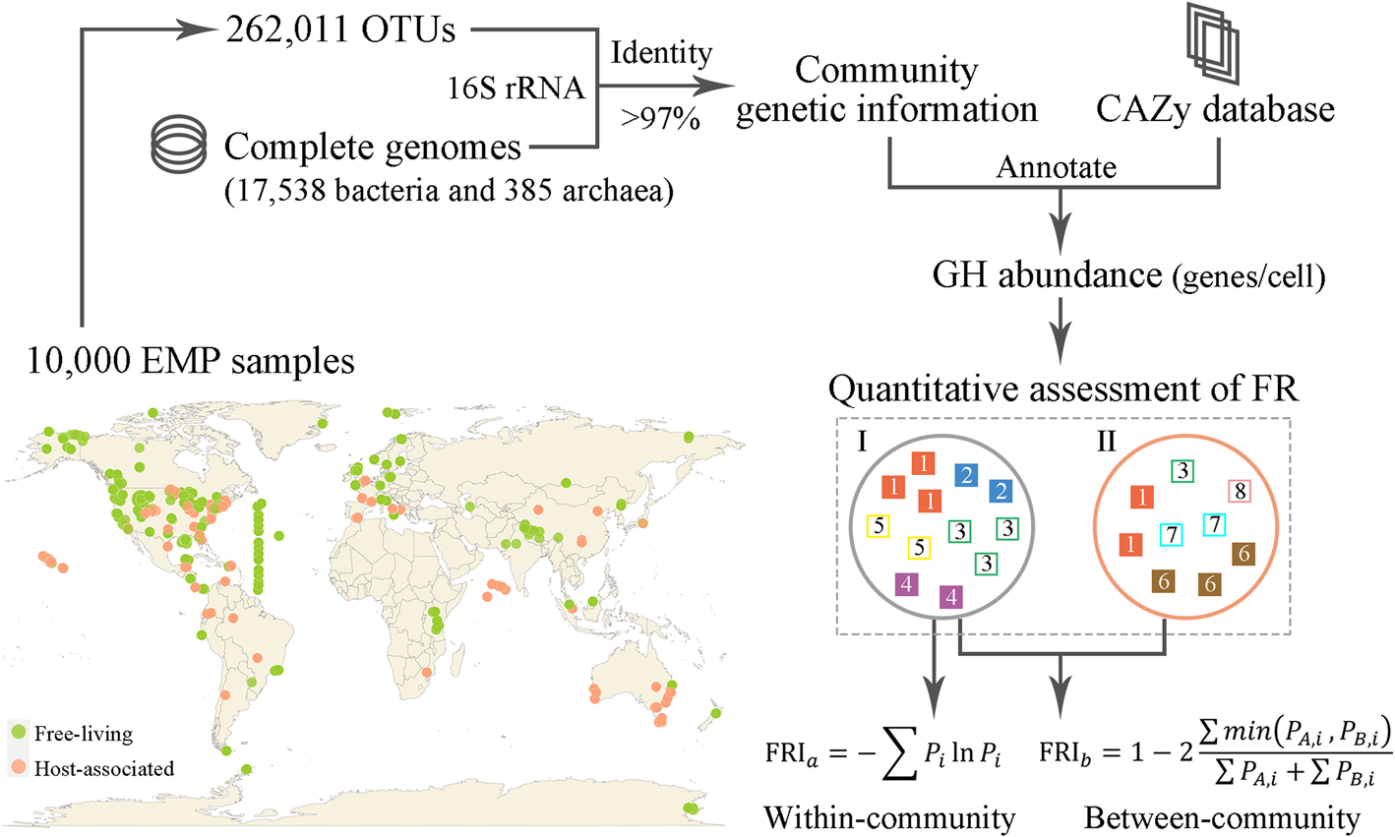
Quantification of functional redundancy within and between prokaryotic communities. OTUs from 10,000 EMP samples were mapped with completely sequenced prokaryotic genome genetic information from the RefSeq database (17,538 bacteria and 385 archaea). These, combined with glycoside hydrolases (GH) described in the CAZy database, were used to calculate gene abundance and quantitatively assess the within-community (FRI_a_) and between-community (FRI_b_) functional redundancy index (“Methods” section). I and II represent different communities, each square indicates a cell, and each number (same color) represents a specific taxon. Filled squares represent cells that can encode a certain function, while hollow squares represent cells that cannot perform that function

Using the Earth Microbiome Project (EMP) data and the prokaryotic genomes from public databases, we previously demonstrated that the sequenced proportion of prokaryotic genomes in global major prokaryotic communities has reached a high level [34, 54]. To correlate community compositions with sequenced genomes and to obtain genetic information about prokaryotic communities, we retrieved the 16S rRNA gene sequences from 10,000 EMP samples and identified the corresponding taxonomic groups of sequenced genomes (Fig. 1). Then, the abundance of GH genes carried by each OTU in the sample was calculated by combining the number of GH genes encoded by prokaryotic genomes and the abundance of corresponding OTU cells.

For a specific metabolic function, we proposed two methods to quantify FR in two aspects: within communities and between communities (Fig. 1 and **“**Methods” section). To evaluate the degree of FR for the coexisting but taxonomically distinct prokaryotic taxa within a community that encodes the same functions, we defined the FRI_a_ value based on the Shannon index. A high FRI_a_ value means a high degree of within-community FR, and 0 indicates that the function is not redundant in a community. To evaluate the taxonomic differences of prokaryotes encoding the same functions between communities, we defined the FRI_b_ value based on the Bray–Curtis index. A high FRI_b_ value indicates a high degree of between-community FR; 1 indicates that the function in two compared communities is encoded by completely different taxa, and 0 means that the function is encoded by the same taxa, i.e., the function is not redundant.

### Functional redundancy of glycoside hydrolase within communities

**Fig. 2:**
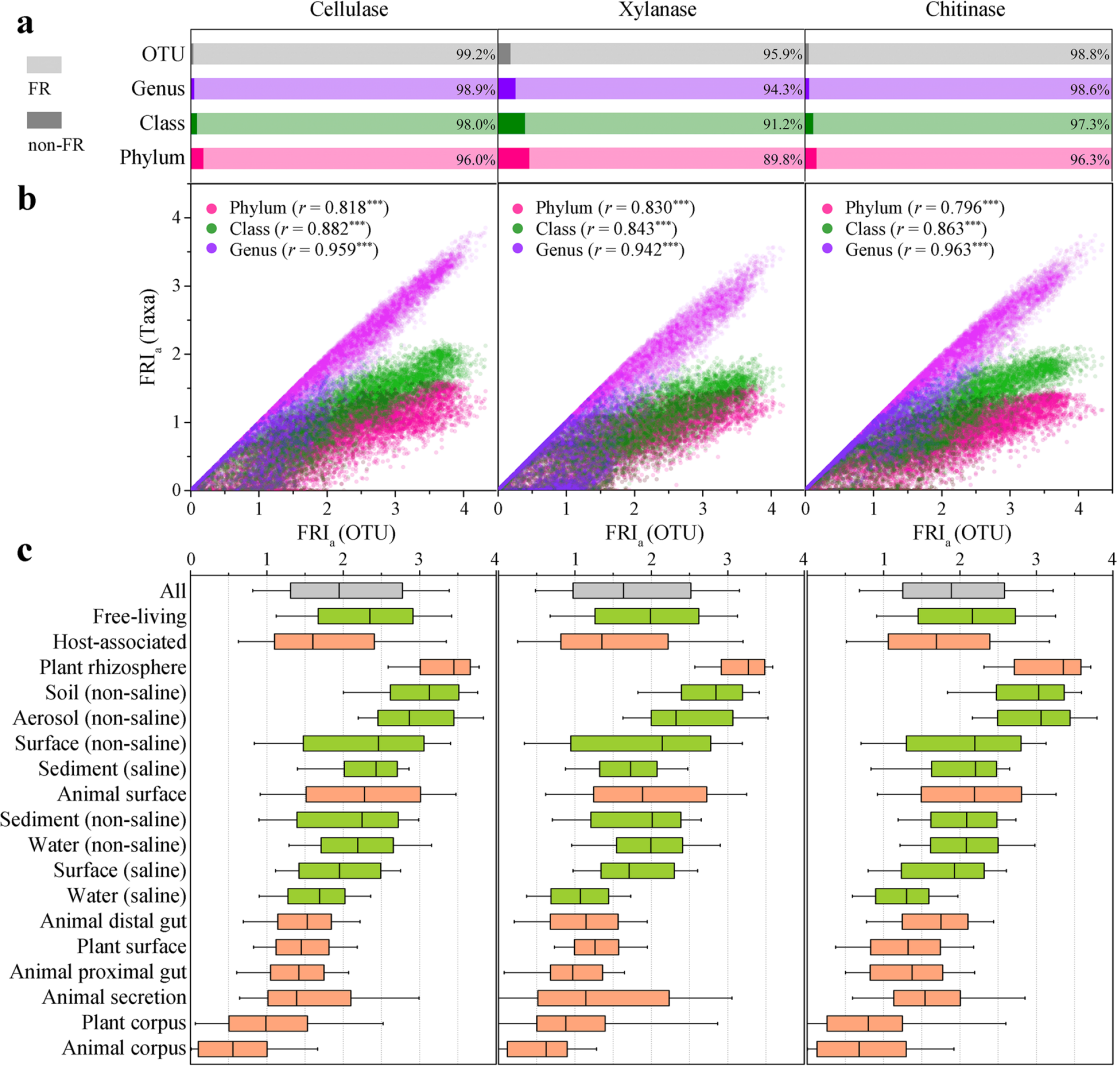
Functional redundancy of glycoside hydrolases is widespread within the community. **a** Multiple coexisting but taxonomically distinct (regardless of the taxonomic level) prokaryotes encoding the same metabolic functions within a community. For cellulases, xylanases, and chitinases, the proportions of functionally redundant communities (light gray) or not (dark gray) are shown at the phylum, class, genus, and OTU levels, respectively. **b** The within-community functional redundancy index FRI_a_ calculated based on OTUs was highly positively correlated with values calculated based on group, subgroup, or genus (Pearson *r*, *P* < 0.001). Each dot represents a community. **c** Quantitative assessment of the degree of functional redundancy within the community of cellulases, xylanases, and chitinases in various environments. A high FRI_a_ value means a high degree of within-community functional redundancy, and 0 indicates that the function is not redundant in the community. The EMPO classified 17 microbial environments as free-living or host-associated. The hypersaline (saline) environment was excluded due to few samples. Orange represents host-associated, and green represents free-living. For the box plots, the middle line indicates the median, the box represents the 25th–75th percentiles, and the error bar indicates the 10th–90th percentiles of observations

According to the FRI_a_ values, almost all prokaryotic communities had the functional potential to encode cellulases (99.7%), xylanases (97.8%), and chitinases (99.6%), and these 3 typical GHs were widely functional redundant (Supplementary Data 1). For the prokaryotic communities containing cellulase genes, 99.2% encoded the genes by multiple OTUs, 98.9% by multiple genera, 98.0% by multiple classes, and 96.0% by multiple phyla (Fig. 2a). Similar results were obtained for xylanases and chitinases. Thus, the prokaryotes encoding cellulases, xylanases, or chitinases within a community were mostly completely taxonomically distinct at different taxonomic levels. This suggested that convergent evolution, rather than phylogenetic conservatism, was responsible for functional redundancy. The FRI_a_ values calculated by OTU were highly positively correlated with those calculated by other taxonomic levels (*P* < 0.001) and with similar trends (Fig. 2b). The subsequent analysis of FR was calculated by OTUs.

For the analyzed EMP samples, the median FRI_a_ values were 1.95 (1.31–2.77) for cellulases, 1.63 (0.98–2.52) for xylanases, and 1.89 (1.25–2.58) for chitinases (Fig. 2c). Pairwise differences in FRI_a_ values of the three enzymes were significant (Wilcoxon Rank Test, *P* < 0.001), indicating that within-community FR was determined by the considered functions. The EMP ontology (EMPO) classifies microbial environments as free-living and host-associated, with further subdivision into 17 environment types [34]. The median FRI_a_ value of cellulases in free-living communities was 2.35 (1.67–2.91) but was only 1.60 (1.10–2.41) in host-associated communities, and the difference between the two was significant (Wilcoxon rank test, *P* < 0.001). Similarly, the degree of FR for xylanases or chitinases in free-living communities was significantly higher than that in host-associated communities. The degree of FR differed greatly among the environmental types. For example, the median FRI_a_ values of cellulases in the soil (non-saline), water (saline), and animal corpus were 3.13, 1.69, and only 0.56, respectively. Therefore, the degree of FR within a community was also determined by the environment type.

**Fig. 3:**
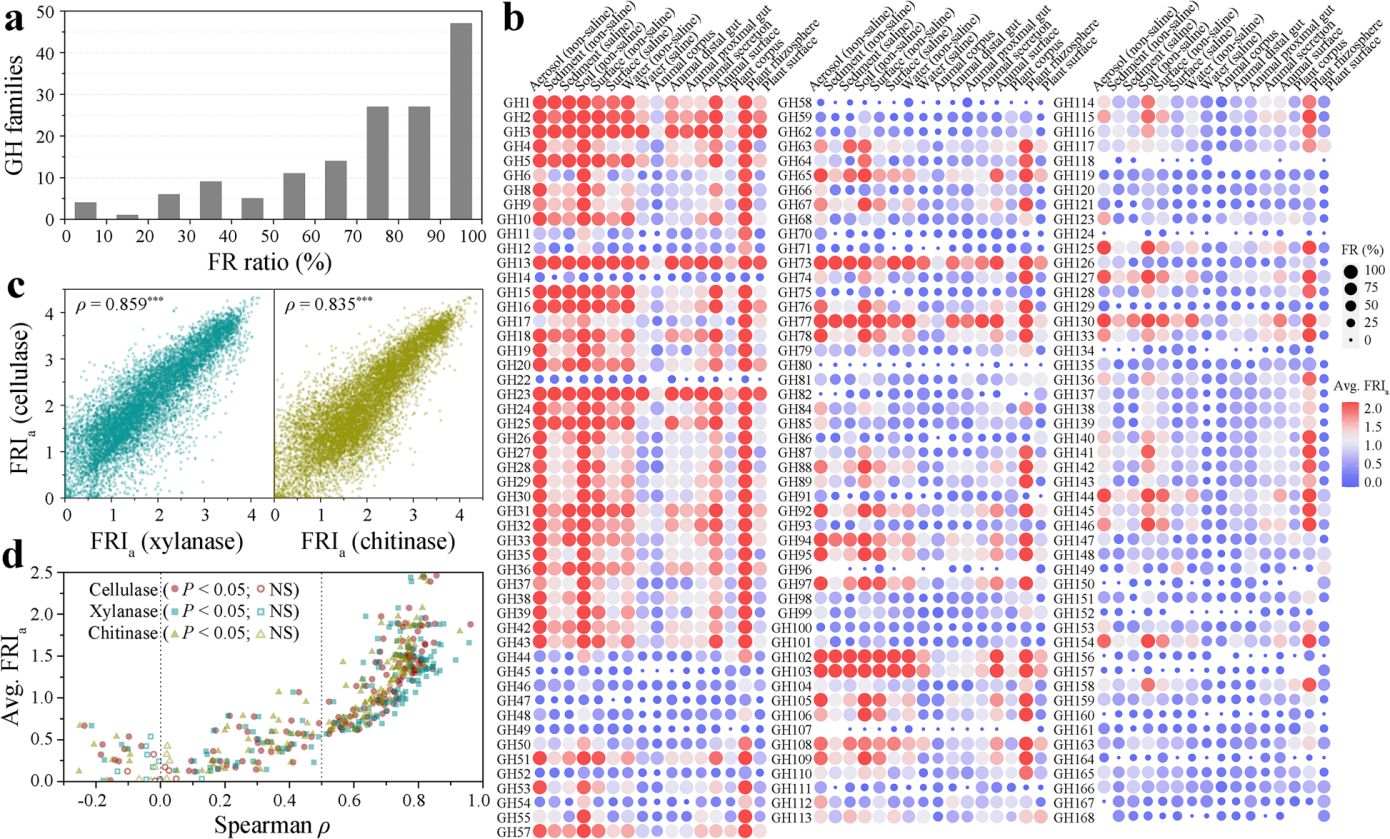
Within-community functional redundancy is largely determined by the type of environment and the functions considered. **a** Statistics on the proportion of all GH families with functional redundancy in global prokaryotic communities. The percentage of functionally redundant communities in the total number of communities encoding that family was calculated for each family. **b** Distribution of FRI_a_ values for 151 GH families in 16 environments. Colors ranging from blue to red represent average FRI_a_ values from low to high. The circle size represents the percentage of functionally redundant communities of each GH family in a certain environment. **c** Significant positive correlation between the FRI_a_ value of cellulases and those of xylanases and chitinases (Spearman *ρ*, *P* < 0.001). Dots represent individual communities. **d** Significant positive correlation between FRI_a_ values of most GH families and cellulases, xylanases, or chitinases (Spearman *ρ*, *P* < 0.05). Higher average FRI_a_ values of GH families tend to have stronger positive correlations. Each symbol represents a family, filled symbols represent significant correlations (*P* < 0.05), and hollow symbols represent non-significant correlations (NS, *P* > 0.05)

In addition to the 3 typical GHs, we also analyzed the FR of each GH family in prokaryotic communities. We identified 152 GH families in the EMP samples, with the exception of GH122, which was excluded because it existed in only 2 samples, and the other 151 analyzed GH families appeared in at least 125 samples (Supplementary Data 2). The results showed that FR existed in all GH families, but the degree of FR varied greatly (Fig. 3a). Seventy-four GH families showed FR in at least 80% of the communities with this family, 126 GH families showed FR in at least 50% of the communities, and only 5 GH families showed FR in less than 20% of the communities. The average FRI_a_ values of GH3, GH23, GH13, and GH77 were more than 2, while those of GH80, GH58, GH107, and GH124 were less than 0.1. The degree of FR (measured by average FRI_a_) varied by more than twofold across different environments for each GH family, and 41 families differed by more than tenfold (Fig. 3b). Therefore, the 151 GH families all showed within-community FR, the extent of which was largely determined by the environment type and the functions considered.

Interestingly, among 16 different environmental types, 101 GH families had the highest average FRI_a_ value in the plant rhizosphere, while 73 GH families had the lowest average FRI_a_ value in the animal corpus (Fig. 3b). These results indicated that different GH families had similar trends in the degree of FR within a community across environments. Furthermore, we found that the FRI_a_ values of cellulases and xylanases (Spearman *ρ* = 0.859, *P* < 0.001), cellulases and chitinases (Spearman *ρ* = 0.835, *P* < 0.001) were highly positively correlated (Fig. 3c). Similarly, the FRI_a_ values of cellulases, xylanases and chitinases were positively correlated with the FRI_a_ values of 135, 137, and 135 GH families, respectively (*P* < 0.001), and Spearman correlation values with 101, 102, and 99 GH families exceeded 0.5 (Fig. 3d). The higher the average FRI_a_ value of the GH family, the stronger the positive correlation between the FRI_a_ values of the GH family and cellulases, xylanases or chitinases. Thus, although the degree of FR of GH within a community varies depending on the environment type and the specific function, it might also be influenced by some common factors.

### Factors influencing within-community functional redundancy

**Fig. 4:**
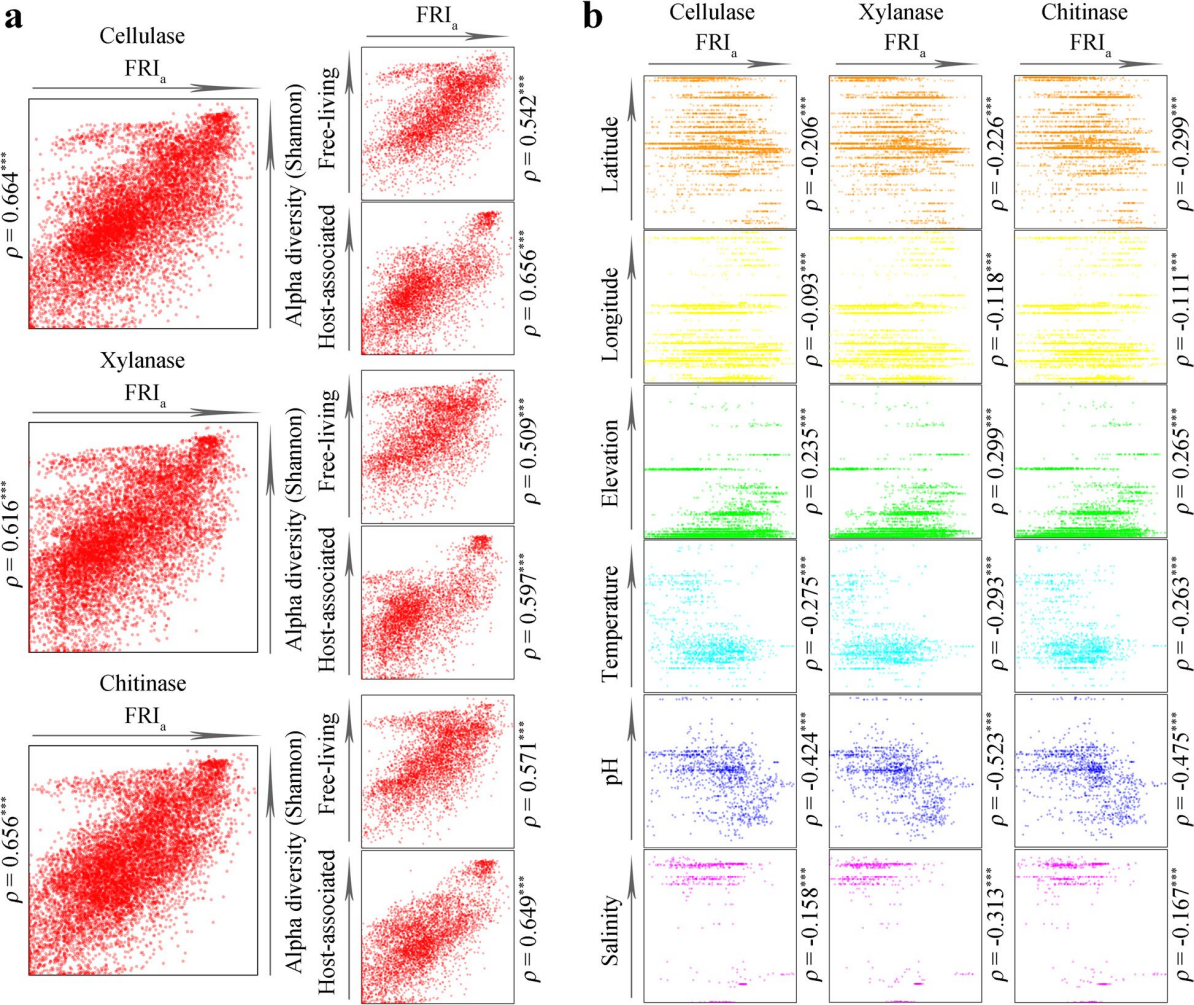
Alpha diversity and various environmental factors affect within-community functional redundancy. **a** On the global scale, in free-living communities and host-associated communities, FRI_b_ values are highly positively correlated with alpha diversity (represented by the Shannon index), taking cellulase, xylanase, and chitinase functions as examples (Spearman *ρ*, *P* < 0.001). **b** In free-living communities, multiple environmental factors, including latitude, longitude, elevation, temperature, pH, and salinity, significantly affect the FRI_a_ values of the three enzymes (Spearman *ρ*, *P* < 0.001). Each dot represents a community

For the 3 typical glucoside hydrolases, cellulases (Spearman *ρ* = 0.664, *P* < 0.001), xylanases (Spearman *ρ* = 0.616, *P* < 0.001), and chitinases (Spearman *ρ* = 0.656, *P* < 0.001) were significantly positively correlated with the Shannon index (Fig. 4a). The FRI_a_ values of the 3 enzymes were also significantly positively correlated with the number of observed OTUs, Chao1 index and Faith’s PD value in the community (*P* < 0.001, Supplementary Figure S1). Although the community alpha diversity of the host-associated environments was significantly lower than that of the free-living environments, they were still positively correlated with the FRI_a_ values of the 3 enzymes. Thus, the diversity of prokaryotic taxa directly affected the degree of FR within a community.

We examined the influences of environmental factors on the FR degree of cellulases, xylanases, and chitinases within a community, including latitude, longitude, elevation, temperature, pH, and salinity (Fig. 4b). We found that all these factors could significantly affect the FRI_a_ values of the 3 enzymes in free-living communities (*P* < 0.001). Under non-extreme conditions, low-latitude, low-longitude, high-elevation, low-temperature, low-pH, or low-salinity environments could enhance the degree of FR within a community for the 3 enzymes. We further conducted a multiple regression analysis on 242 free-living communities possessing all the above factors. The results showed that the variation in the degree of FR within a community for cellulases (adjusted *R*^2^ = 0.716), xylanases (adjusted *R*^2^ = 0.578), and chitinases (adjusted *R*^2^ = 0.633) could be largely explained by the combined effects of community diversity (represented by the Shannon index), latitude, longitude, elevation, temperature, pH, and salinity.

**Fig. 5:**
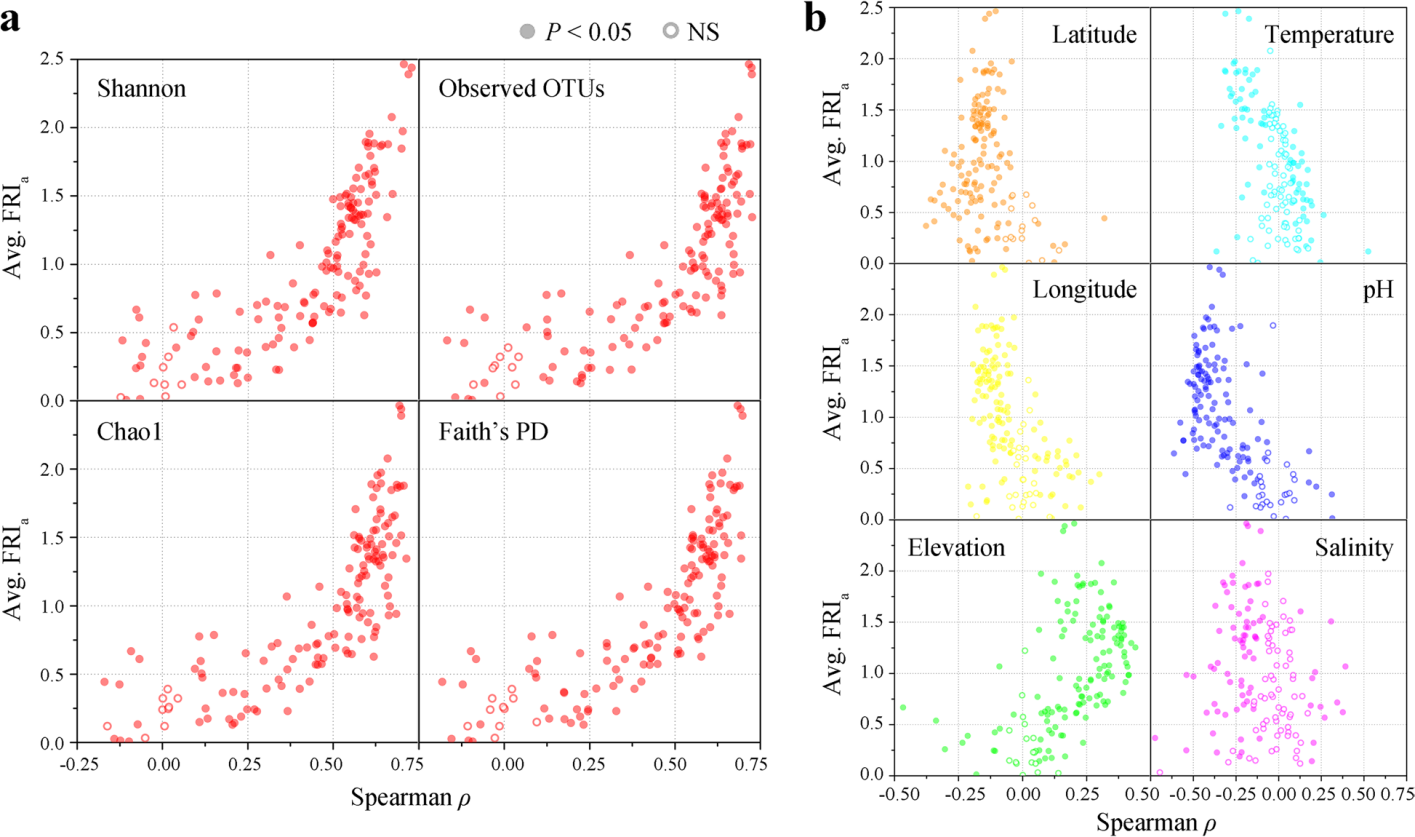
Influencing factors of within-community functional redundancy for 151 GH families. **a** Spearman correlation between FRI_a_ values of 151 GH families and alpha diversity. Families with higher average FRI_a_ values tend to have stronger positive correlations with alpha diversity. Alpha diversity indices include observed OTUs, Shannon index, Chao1 index, and Faith’s PD value. **b** Spearman correlations between six environmental factors and FRI_a_ values of each GH family. Environmental factors include latitude, longitude, elevation, temperature, pH, and salinity. Each dot represents a family, filled circles represent significant correlations (*P* < 0.05), and hollow circles represent non-significant correlations (NS, *P* > 0.05)

For the analyzed 151 GH families, the FRI_a_ values of 134, 134, 134, and 133 GH families were significantly positively correlated with the Shannon index, observed OTUs, Chao1 index and Faith’s PD value, respectively (*P* < 0.001, Fig. 5a). The Spearman correlation values of 79, 91, 90, and 85 GH families exceeded 0.5, respectively. It is noteworthy that the higher the average FRI_a_ value of the GH family, the stronger the positive correlation of the FRI_a_ value with community diversity. These results suggested that the within-community FR degrees of most GH families were affected by community diversity and that the families with higher within-community FR were more affected. We also individually analyzed the correlations of latitude, longitude, elevation, temperature, pH, and salinity with the FRI_a_ value of each GH family (Fig. 5b). The results showed that these 6 factors were significantly correlated with the FRI_a_ values of 137, 126, 133, 78, 124, and 78 GH families, respectively. Hence, the FR degrees of most GH families within a community were collectively influenced by community diversity and the six factors, and the contribution of community alpha diversity was the largest.

### Functional redundancy of glycoside hydrolases between communities

**Fig. 6:**
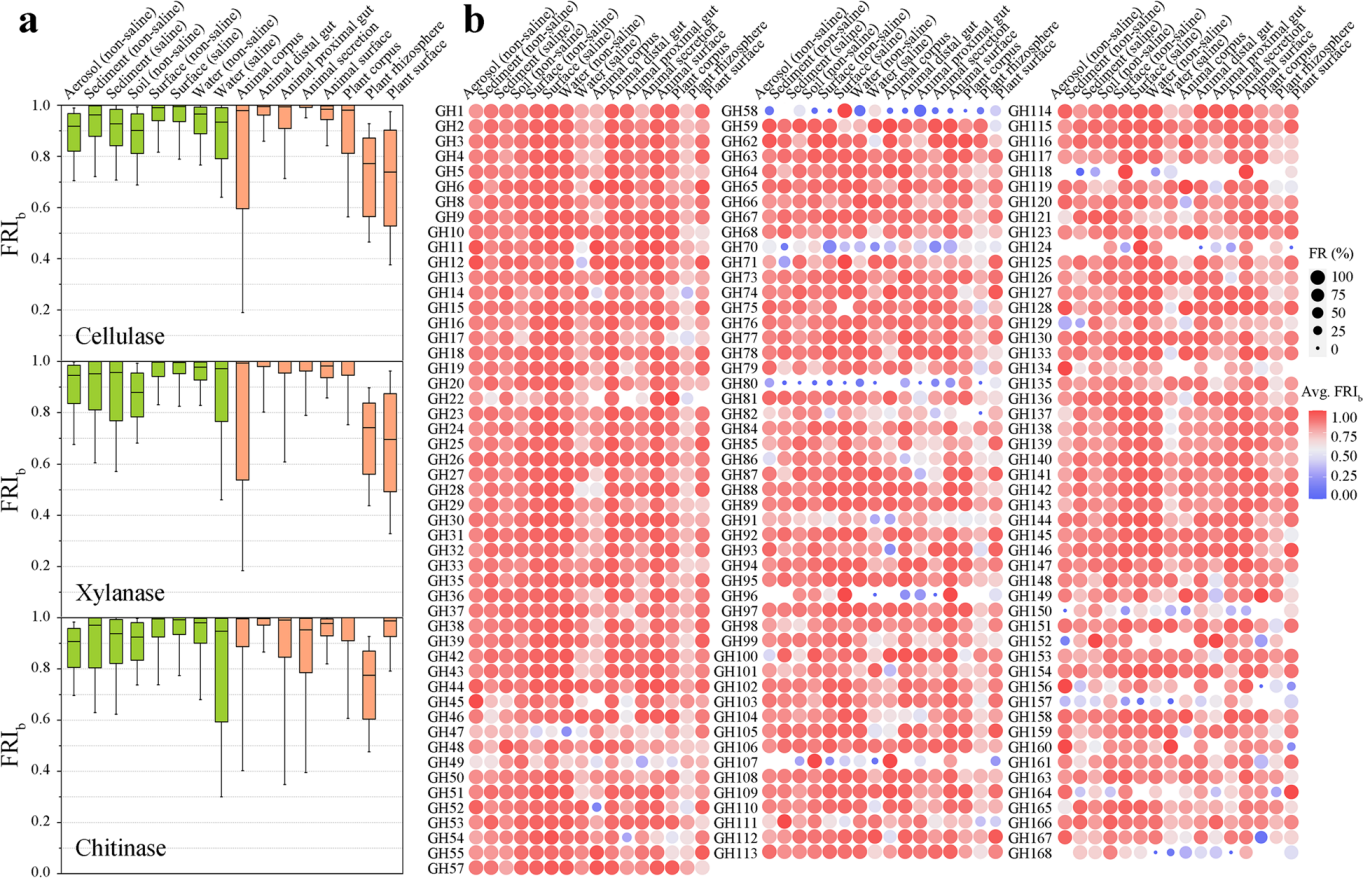
The identity of prokaryotic taxa encoding each function varies substantially across communities. **a** Cellulases, xylanases, and chitinases share a high degree of between-community functional redundancy in different environmental types. A high FRI_b_ value indicates a high degree of between-community functional redundancy, and 0 indicates that the function is not redundant. Orange represents host-associated, and green represents free-living. For the box plots, the middle line indicates the median, the box represents the 25th–75th percentiles, and the error bar indicates the 10th–90th percentiles of observations. **b** Distribution of FRI_b_ values for 151 GH families in 16 environments. Colors ranging from blue to red represent average FRI_b_ values from low to high. The circle size represents the percentage of between-community functional redundancy of the family in a specific environment

Based on the FRI_b_, we calculated the degree of FR for cellulases, xylanases, and chitinases between prokaryotic communities of different environment types (Fig. 6a and Supplementary Data 3). We found that in the 16 analyzed environmental types, cellulases, xylanases, and chitinases showed between-community FR in at least 99.7% (animal corpus), 98.3% (animal corpus), and 99.3% (plant corpus) of prokaryotic communities. Moreover, the median FRI_b_ values of cellulases, xylanases, and chitinases were more than 0.772 (plant rhizosphere), 0.696 (plant surface), and 0.775 (plant rhizosphere), respectively. Therefore, cellulases, xylanases, and chitinases all showed a high degree of FR between communities in different environmental types. Interestingly, among different environmental types, the median FRI_a_ values of cellulases (Spearman *ρ* =  − 0.526, *P* = 0.036), xylanases (Spearman *ρ* =  − 0.622, *P* = 0.010), and chitinases (Spearman *ρ* =  − 0.671, *P* = 0.004) were significantly negatively correlated with the median FRI_b_ values (Supplementary Figure S2). These results indicated that the FRI_a_ and FRI_b_ values showed an opposite trend, and that communities with higher within-community FR had lower between-community FR.

The 151 GH families were analyzed for FR between communities in each of the 16 environment types, which were divided into a total of 2345 groups of “GH family-environment type” (Fig. 6b and Supplementary Data 4). In 84.8% of the “GH family-environment type” groups, at least 90% of the prokaryotic community pairs were functionally redundant, while in only 2.9% of the groups, less than 50% of the prokaryotic community pairs had FR. Each GH family had an average FRI_b_ value of more than 0.7 in at least one environmental type. Furthermore, the differences in the average FRI_b_ values of all GH families were more than 1.2 times under different environments; 52 GH families with the lowest average FRI_b_ values were in the plant rhizosphere, and 37 GH families with the highest average FRI_b_ values were in the surface (saline), and this was opposite of the result for the FRI_a_ values. These results indicated that between-community FR was common for all GH families, that is, the taxonomic groups encoding the same functions were highly different between communities and showed an opposite trend to FRI_a_ values in different environment types.

### Factors influencing between-community functional redundancy

**Fig. 7:**
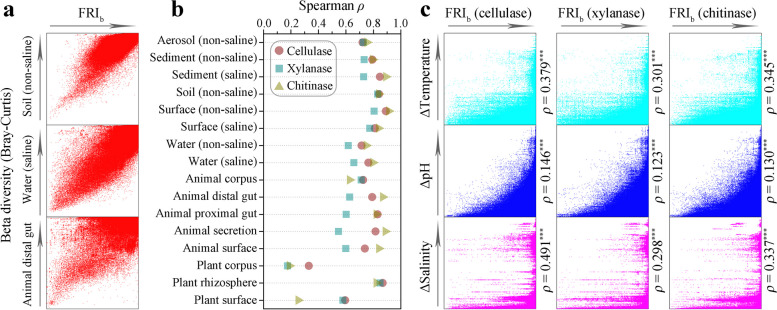
Beta diversity and various environmental factors affect the degree of between-community functional redundancy. **a** Similar relationship between FRI_b_ values of cellulases and beta diversity (characterized by the Bray–Curtis index) in highly structured soil (non-saline), freely diffusing water (saline), and host-affected animal distal gut. **b** FRI_b_ values of cellulases, xylanases, and chitinases in 16 different environments significantly positively correlated with beta diversity (Spearman *ρ*, *P* < 0.001). **c** Differences in environmental factors between communities, including Δtemperature, ΔpH, and Δsalinity, significantly affect the FRI_b_ values of the three enzymes in free-living communities (Spearman *ρ*, *P* < 0.001). Each dot represents a set of comparisons between two communities

To assess factors that might influence the degree of between-community FR of GH, we hypothesized that, similar to the relationship of the FRI_a_ value and alpha diversity, the FRI_b_ value might be related to the beta diversity of prokaryotic communities. Our analysis showed that in the highly structured soil (non-saline) (Spearman *ρ* = 0.840, *P* < 0.001), freely diffused water (saline) (Spearman *ρ* = 0.762, *P* < 0.001) or host-affected animal distal gut (Spearman *ρ* = 0.790, *P* < 0.001), the FRI_b_ values of cellulases were highly positively correlated with beta diversity (Fig. 7a, represented by the Bray–Curtis index). We further determined that the FRI_b_ values of cellulases, xylanases, and chitinases were all significantly positively correlated with the beta diversity in the 16 different environmental types (*P* < 0.001, Fig. 7b). This suggested that greater differences in the compositions of two prokaryotic communities might cause greater differences in the taxonomic groups encoding these three enzymes.

We also explored the effects of temperature, pH, or salinity differences on the between-community FRI_b_ values of cellulases, xylanases, and chitinases (Fig. 7c). The results showed that the Δtemperature, ΔpH, and Δsalinity values were positively correlated with the FRI_b_ values of the three enzymes, respectively (*P* < 0.001). Differences in the taxonomic compositions encoding these three enzymes increased with differences in temperature, pH, and salinity values of the two communities. Thus, the degree of between-community FR for GH was significantly affected by at least beta diversity, Δtemperature, ΔpH, and Δsalinity, and the effect of beta diversity was the strongest.

### Quantifying functional redundancy of glycoside hydrolases based on metagenomes

**Fig. 8:**
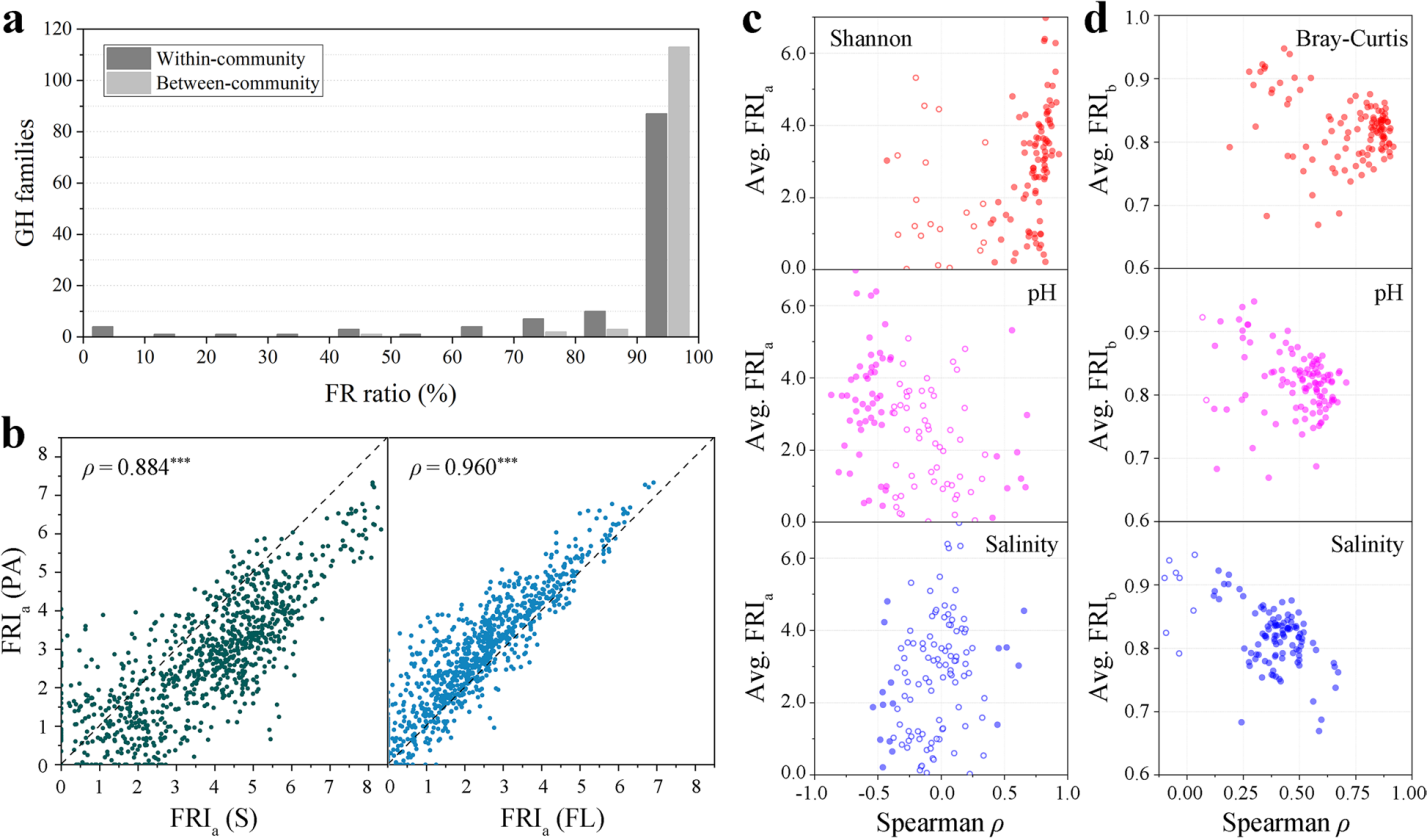
Functional redundancy of glycoside hydrolases in metagenomes. **a** Statistics on the proportion of all GH families with functional redundancy in metagenomes from the Yellow River Delta, China. The percentage of functionally redundant communities in the total number of communities encoding that family was calculated for each family. **b** Significant positive correlation (Spearman *ρ*, *P* < 0.001) between the FRI_a_ values of GHs in sediment (S) benthic communities and those in particle-associated (PA) planktonic communities or free-living (FL) planktonic communities. Each dot represents the FRI_a_ value of an individual family in a sample. **c** Spearman correlation between FRI_a_ values of each GH family and influencing factors (Shannon indices, pH, and salinity) in all of the samples. **d** Spearman correlation between FRI_b_ values of each GH family and influencing factors (Bray–Curtis indices, ΔpH, and Δsalinity) in all of the samples. Each dot represents a family, filled circles represent significant correlations (*P* < 0.05), and hollow circles represent non-significant correlations (NS, *P* > 0.05)

Recently, metagenomic data have been employed to illustrate habitat-specific functional redundancy in prokaryotic communities [13]. Utilizing metagenomic data collected from the Yellow River Delta in China, we quantified the FR of GHs in sediment (S) benthic communities, particle-associated (PA) planktonic communities, and free-living (FL) planktonic communities. The results revealed broad functional redundancy both within and between these prokaryotic communities (Fig. 8a). In comparison to EMPO, the FR degrees of GHs in the S communities were significantly correlated with those of the sediment (saline) environments (Spearman *ρ* = 0.810, *P* < 0.001). Similarly, the FL communities exhibited a strong correlation with the water (saline) environments (Spearman *ρ* = 0.736, *P* < 0.001), while the PA communities were most related to the surface (saline) environments (Spearman *ρ* = 0.813, *P* < 0.001). The FRI_a_ values of the three community types were highly positively correlated, yet distinct habitat differences were evident: the S communities displayed the highest FRI_a_ value, followed by the PA communities, and the FL communities had the lowest value (Fig. 8b).

Consistent with our EMP results, the degrees of FR in both benthic and planktonic communities were significantly influenced by community alpha diversity and environmental factors. Most GH families exhibited a notably positive correlation (*P* < 0.001) between their FRI_a_ values and alpha diversity indices, pH, and salinity within communities (Fig. 8c). Similarly, the FRI_b_ values of most GH families were significantly positively correlated (*P* < 0.001) with community beta diversity indices, ΔpH and Δsalinity (Fig. 8d). Hence, the habitat specificity of functional redundancy, as depicted by metagenomic data, was a combined outcome of community diversity, environmental factors, and other influencing factors.

## Discussion

Recent multitude studies on environments, from soil to ocean and to human gut, suggested that multiple taxonomically distinct microorganisms capable of performing similar metabolic functions coexist in prokaryotic communities, and FR appears to be a common aspect of many microbial systems [8]. Nevertheless, quantitative assessment of FR has not been attempted until recently [13, 22], and they are only for within-community FR. Here, we proposed two quantitative approaches to assess two aspects of FR based on a large-scale analysis of prokaryotic community genetic information at a global scale. On the one hand, the FRI_a_ value is used to quantify the diversity of prokaryotes encoding the same metabolic function within a community. On the other hand, the FRI_b_ value is used to quantify the identity dissimilarities of taxa encoding the same function between communities. These two methods can be widely used to measure the degree of FR for the metabolic functions in various environmental types, helping to determine the FR formation process and influencing factors, and providing new insights into the relationships between prokaryotic community biodiversity and ecosystem functions.

GHs are a large class of enzymes that are key to degrading carbohydrates and affecting the carbon cycle in nature. The major complex polysaccharide-degrading enzymes, such as cellulases, xylanases, and chitinases, have received the most attention [25, 55, 56]. We determined that GHs, not only the typical polysaccharide degradation functions (cellulases, xylanases, and chitinases) but also each of the 151 GH families, are widely functionally redundant within and between prokaryotic communities. That is, each type of GH function is encoded by multiple taxonomically distinct prokaryotes within a community and by highly differentiated taxa between communities. The prokaryotic species with similar genes (functional potentials) tend to compete with each other by occupying the same metabolic niche, and thus, this widely-present FR appears to be inconsistent with the expectation that species should occupy distinct metabolic niches.

It is known that deterministic (e.g., environmental selection and biotic interactions) and stochastic (e.g., speciation, birth, death, and immigration) processes simultaneously remodel prokaryotic community assemblies [57–59]. Our recent research indicated that deterministic processes tend to play a predominant role in free-living and plant-associated environments, whereas stochastic processes are the primary contributors to animal-associated environments [60]. However, unlike community assembly, the assembly of functional genes within communities is predominantly governed by deterministic processes across all environments [60]. The conserved function profile is in sharp contrast to the highly diverse taxa encoding the same function, which highlights the decoupling of prokaryotic community function and taxonomic composition. Environmental conditions can predict community functional traits well, but predictions regarding their taxonomic compositions are notably weak.

FR is generally regarded as an indicator of neutral assembly: within-community FR reflects the quasi-neutral coexistence of competitors within a metabolic niche, and between-community FR results from ecological drift between equivalent organisms [61, 62]. However, we found that the degree of FR is not random, and the redundancy extents of various GH functions within or between communities are mainly determined by the environment types and functions. The FR degree in free-living communities is significantly higher than that in host-associated communities. Furthermore, differences in the FR degree of GHs in various environments, within or between communities, are largely due to the combined effects of community diversity and environmental factors such as pH, temperature, and salinity. In particular, the degrees of within-community FR are most strongly affected by alpha diversity, while the between-community FR degrees are most strongly influenced by beta diversity. All these results suggest that there is a highly positive correlation between FR degree and community diversity and that functions with a higher redundancy degree tend to have a stronger correlation. Hence, the degree of FR is a stable prokaryotic community characteristic that is highly deterministic and influenced by community diversity and environmental factors.

Within-habitat heterogeneity is a driver of animal or plant diversity and is also associated with microbial communities [63–65]. The habitat heterogeneity of free-living communities is generally higher than that of host-associated communities, thus leading to higher community diversity [66]. Highly dispersal environments, such as water, have lower habitat heterogeneity than structural environments, such as soil, and thus also have lower community diversity [67]. High habitat heterogeneity is thought to provide many unique ecological niches, which help to reduce competition and thus preserve high community diversity [68]. Our results indicate that the FR of prokaryotic communities is closely related to their habitats, and the highly positive correlation between the FR degree and community diversity might actually reflect the effects of habitat heterogeneity. Ignoring the niche diversification brought about by habitat heterogeneity can lead to the illusion of functional “redundancy”, while those functionally “redundant” taxa may actually play an integral role. Thus, functional redundancy should be seen as a community characteristic as well as a manifestation of the community diversity of prokaryotes capable of a particular metabolic function.

## Conclusions

Functional redundancy widely exists in prokaryotic communities in different environments, e.g., soil, ocean, and human. However, it is unclear the degree of functional redundancy, its formation process, and influencing factors in various environments. Here, based on a large-scale analysis of prokaryotic community genetic information at a global scale, we proposed two methods to respectively quantify the degree of functional redundancy within and between communities and applied them to the analysis of carbohydrate degradation. Our results suggested that various glycoside hydrolase functions showed broad functional redundancy; that is, the same function was encoded by multiple taxonomically distinct prokaryotes within a community and by taxa that were highly distinct between communities. The degree of functional redundancy was highly affected by community diversity and environmental factors (e.g., pH, temperature, and salinity), for both within-community and between-community. The degree of functional redundancy is not determined by random factors but is highly deterministic. Functional redundancy should be regarded as a stabilized community characteristic as well as a manifestation of the community diversity of prokaryotes capable of a particular metabolic function. These results promote our understanding of the relationships between prokaryotic community biodiversity and ecosystem functions.

### Supplementary Information

Supplementary file 1. Supplementary Figure S1. Alpha diversity affects the degree of within-community functional redundancy. The FRI_a_ values of cellulases, xylanases and chitinases within the community are significantly positively correlated with observed OTUs (number of unique tag sequences), Chao1 index, and Faith’s PD value, respectively (Spearman *ρ*, *P* < 0.001). Dots in red represent individual communities.

Supplementary file 2. Supplementary Figure S2. Communities with higher within-community functional redundancy have lower between-community functional redundancy. The FRI_a_ values of cellulases, xylanases and chitinases are significantly negatively correlated with their FRI_b_ values among different environmental types (Spearman *ρ*, *P < 0.05, **P < 0.01). Each symbol represents an environment.

Supplementary file 3. Supplementary Data 1. The FRI_a_ values of cellulases, xylanases and chitinases in prokaryotic communities.

Supplementary file 4. Supplementary Data 2. Data of the FRI_a_ values for 151 GH families in prokaryotic communities.

Supplementary file 5. Supplementary Data 3. The FRI_b_ values of cellulases, xylanases and chitinases in different environments.

Supplementary file 6. Supplementary Data 4. Average FRI_b_ values for 151 GH families in different environments.

## Data Availability

The main data supporting the findings of this study are available within the article and its Supplementary Information. All other data supporting the findings of this study are available from the corresponding authors upon reasonable request. All of the 16S rRNA amplicon sequencing raw data have been deposited in the NCBI Sequence Read Archive database under the BioProject ID PRJNA657933.

## Abbreviations

CAZy: Carbohydrate-active enzyme
EMP: Earth microbiome project
EMPO: EMP ontology
FR: Functional redundancy
FL: Free-living
GH: Glycoside hydrolase
OTU: Operational taxonomic unit
PA: Particle-associated
S: Sediment
