# Supplementary figures and images for: Quantifying functional redundancy in polysaccharide-degrading prokaryotic communities
Source: Microbiome. 2024 Jul 2;12:120. doi: 10.1186/s40168-024-01838-5 (PMC11218364; doi:10.1186/s40168-024-01838-5)

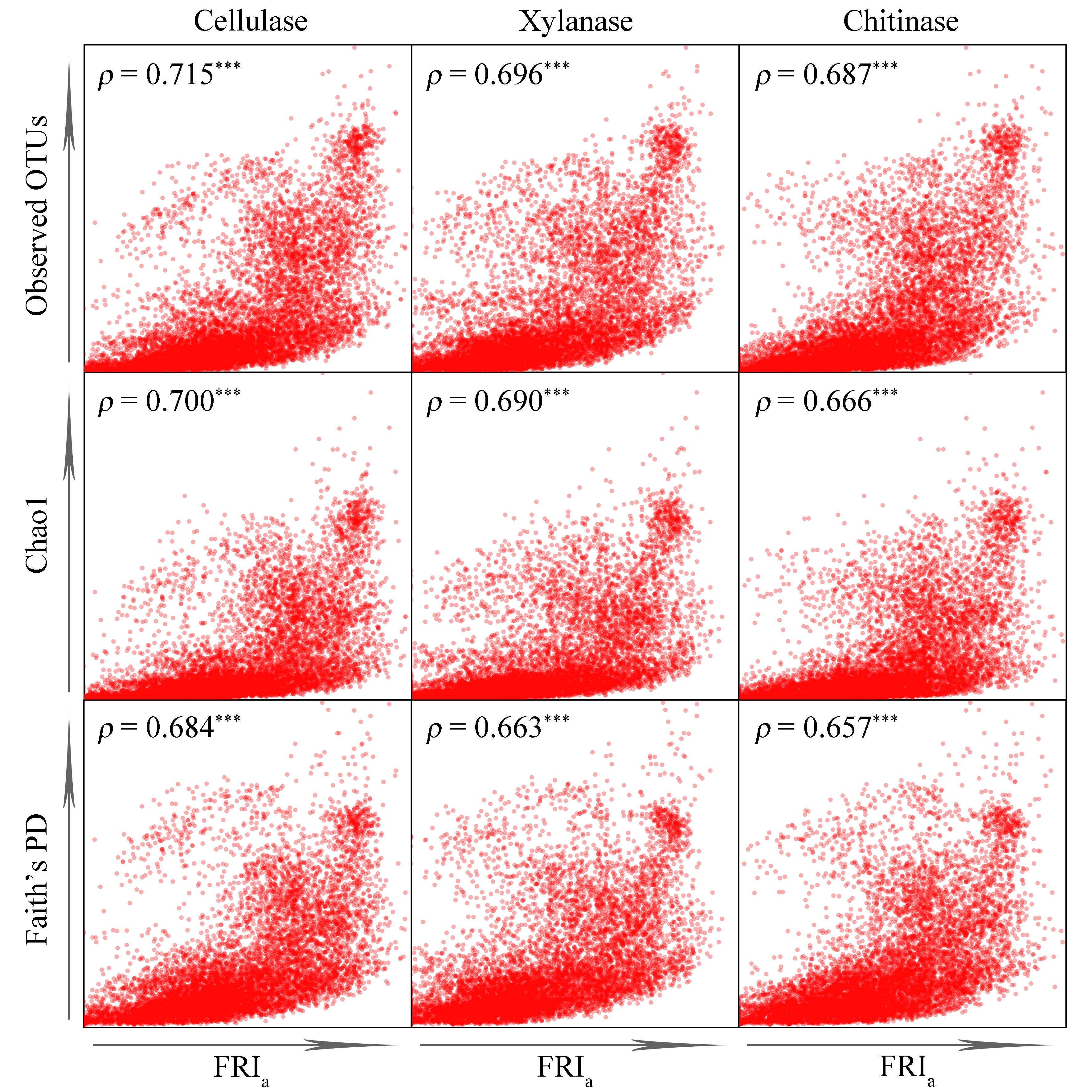

Supplement: Supplementary file 2 — Supplementary file 1. Supplementary Figure S1. Alpha diversity affects the degree of within-community functional redundancy. The FRIa values of cellulases, xylanases and chitinases within the community are significantly positively correlated with observed OTUs (number of unique tag sequences), Chao1 index, and Faith’s PD value, respectively (Spearman ρ, P < 0.001). Dots in red represent individual communities. [file 40168_2024_1838_MOESM1_ESM.tif]

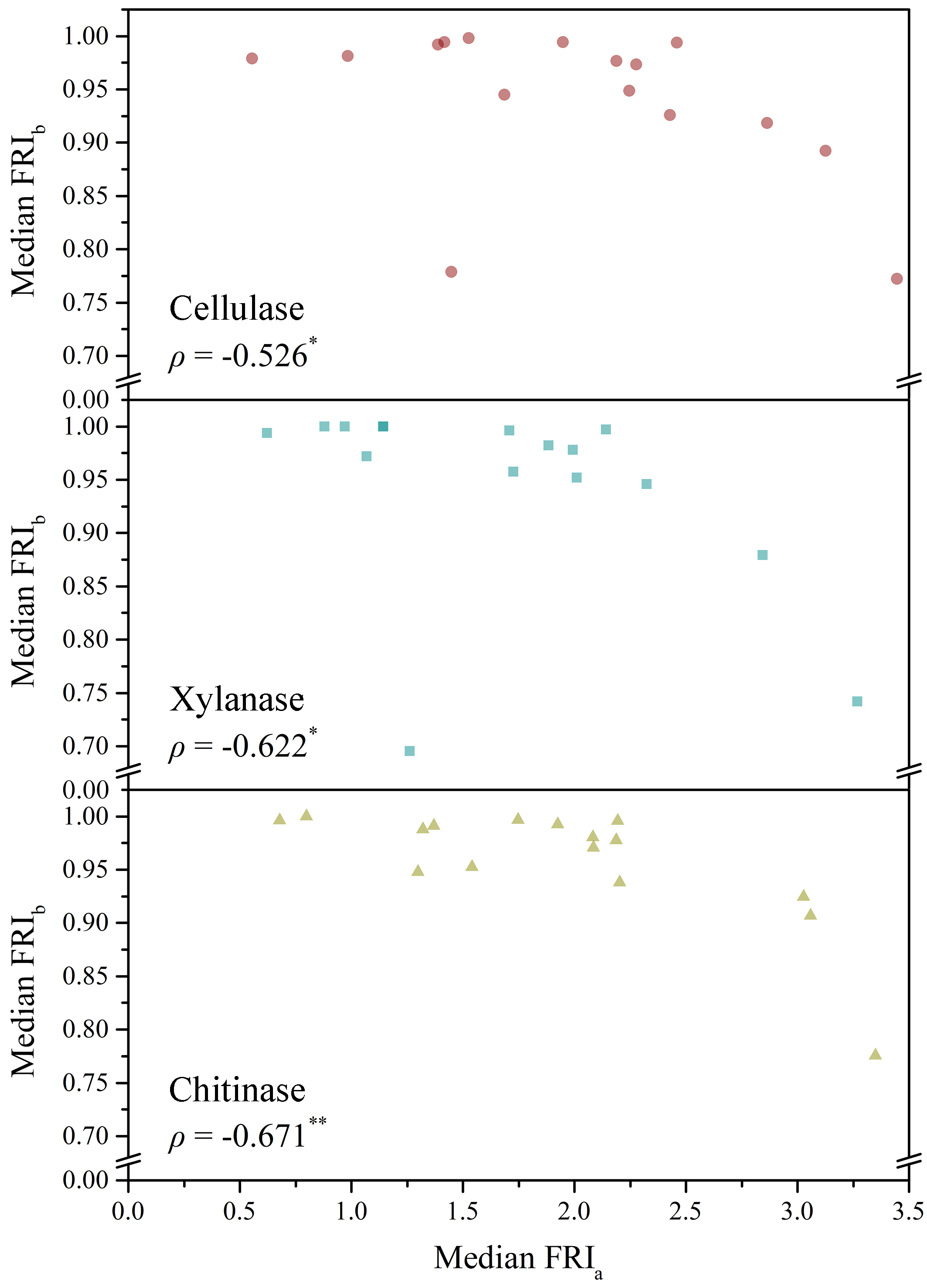

Supplement: Supplementary file 3 — Supplementary file 2. Supplementary Figure S2. Communities with higher within-community functional redundancy have lower between-community functional redundancy. The FRIa values of cellulases, xylanases and chitinases are significantly negatively correlated with their FRIb values among different environmental types (Spearman ρ, *P < 0.05, **P < 0.01). Each symbol represents an environment. [file 40168_2024_1838_MOESM2_ESM.tif]
